# Supplementary material for: Phytochemical Composition, Antibacterial, Antioxidant and Antidiabetic Potentials of Cydonia oblonga Bark
Source: Molecules. 2022 Sep 26;27(19):6360. doi: 10.3390/molecules27196360 (PMC9571850; doi:10.3390/molecules27196360)
Supplement: Supplementary file 1 [file molecules-27-06360-s001.zip › molecules-1931123-supplementary.pdf]

Supplementary Material

Phytochemical Composition, Antibacterial, Antioxidant and Antidiabetic Potentials of *Cydonia oblonga* Bark

Shaymaa Najm Abed [1], Sania Bibi [2,3], Marwa Jan [2], Muhammad Talha [2], Noor Ul Islam [4,*], Muhammad Zahoor [2,*] and Fakhria A. Al-Joufi

[1] Nursing Department, College of Applied Medical Sciences, Jouf University, Sakaka, 42421, Saudi Arabia
[2] Department of Biochemistry, University of Malakand, Chakdara 18800, Pakistan
[3] Department of Microbiology University of Swabi, Swabi 23562, Pakistan
[4] Department of Chemistry, University of Malakand, Chakdara 18800, Pakistan
Department of Pharmacology, College of Pharmacy, Jouf University, Aljouf 72341, Saudi Arabia
* Correspondence: nooruomchem@gmail.com (N.U.I.); mohammadzahoorus@yahoo.com (M.Z.)

GC-MS characterization of purified fractions.

GC-MS analysis of Fraction 1

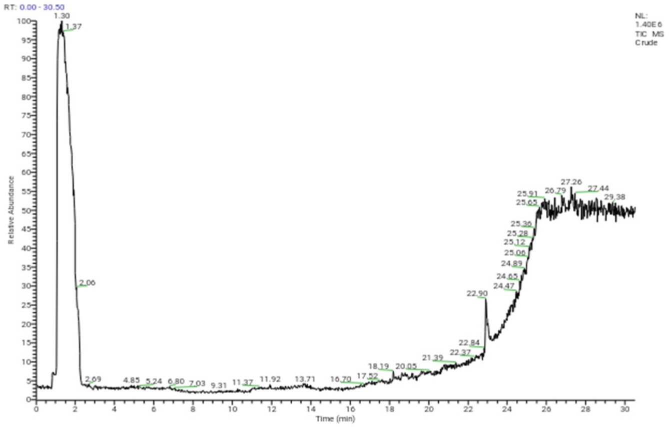

Figure S1. GC-MS chromatogram of fraction 1 of *Cydonia oblonga.*

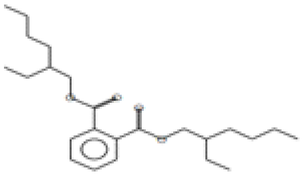

Bis(2-ethylhexyl) phthalate

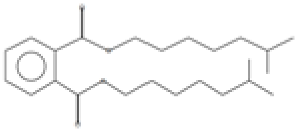

Diisooctyl phthalate

Figure S2. Major phytochemical compounds identified in Fraction 1of *Cydonia oblonga.*

(A)

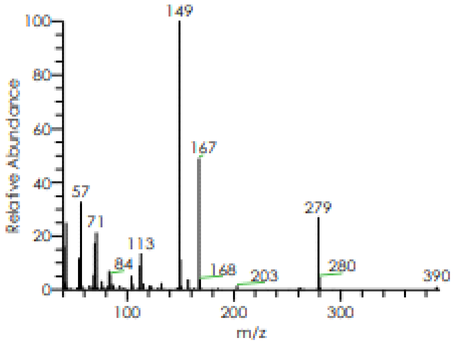

(B)

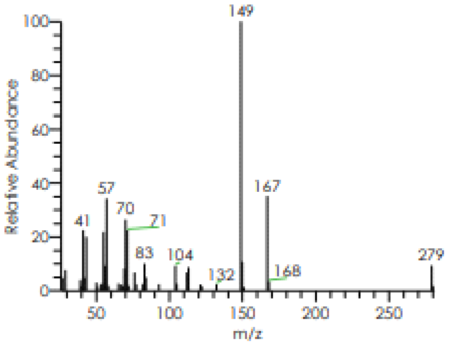

**(A)** Bis(2-ethylhexyl) phthalate	**(B)** Diisooctyl phthalate

Figure S3. Individual mass fragmentation of each compound.

Table S1. List of major components and their parameters identified in methanolic extract of *Cydonia oblonga.*

Retention time (RT)	Molecular mass (g/mol)	Molecular formula	Area %	Area	Peak hight	Compound name
22.90	390	$C_{24}H_{38}O_4$	0.86	513949.22	155477.32	Bis(2-ethylhexyl) phthalate
22.90	390	$C_{24}H_{38}O_4$	0.86	513949.22	155477.32	Diisooctyl phthalate

GC-MS analysis of Fraction 2

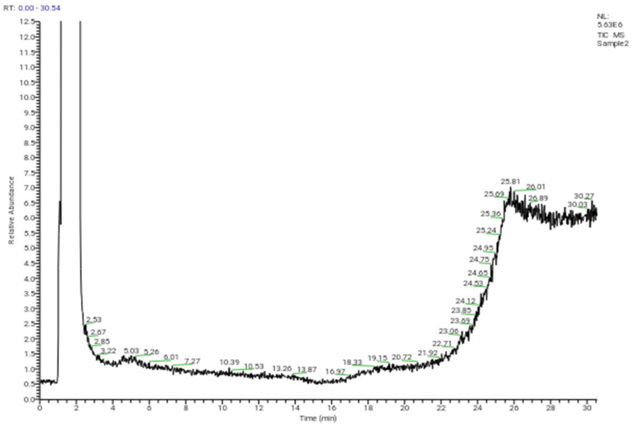

Figure S4. GC-MS chromatogram of Fraction 2 of *Cydonia oblonga.*

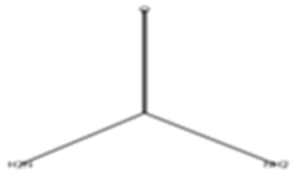

Carbamide

Figure S5. Major phytochemical compounds identified in fraction 2 of Cydonia oblonga.

(A)

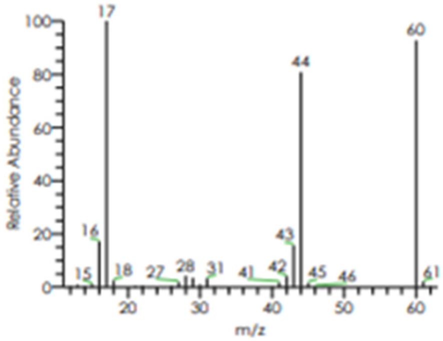

Figure S6. Individual mass fragmentation of compound. (A) Carbamide.

Table S2. List of major components and their parameters identified in Fraction 2 of *Cydonia oblonga.*

Retention time (RT)	Molecular mass (g/mol)	Molecular formula	Area %	Area	Peak hight	Compound name
2.22	60	$C_2H_4O_2$	17.17	17519124 .00	3371034.19	Carbamide

GC-MS analysis of Fraction 3

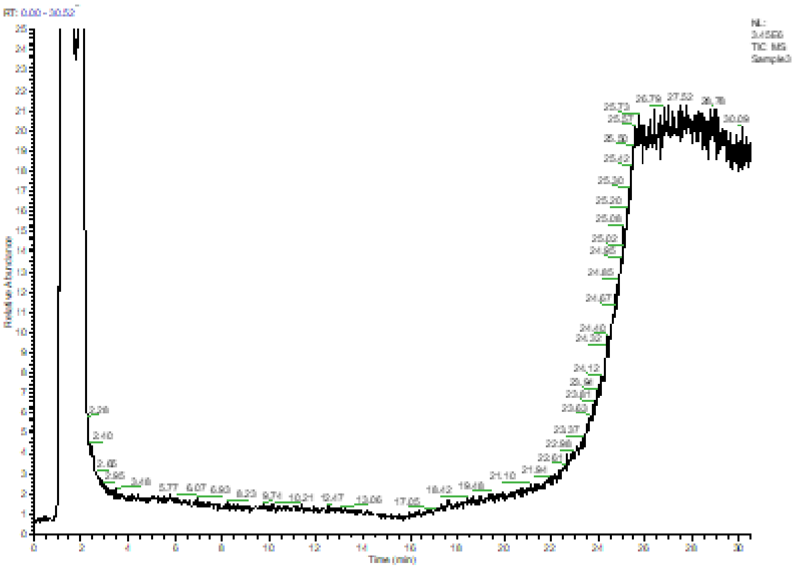

Figure S7. GC-MS chromatogram of Fraction 3 of *Cydonia oblonga.*

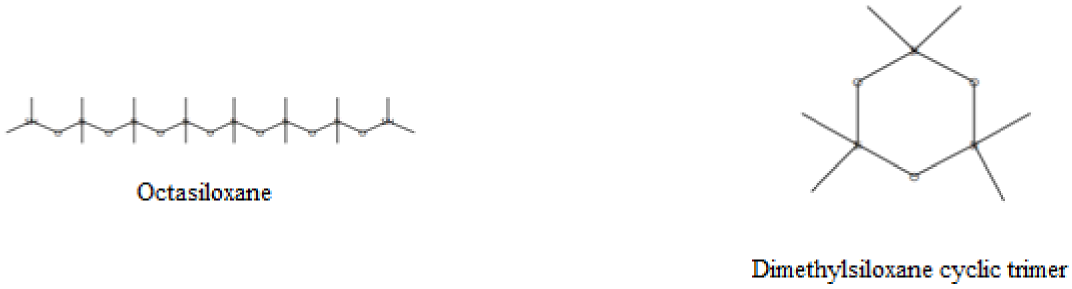

Figure S8 Major phytochemical compounds identified in fraction 3 of *Cydonia oblonga.*

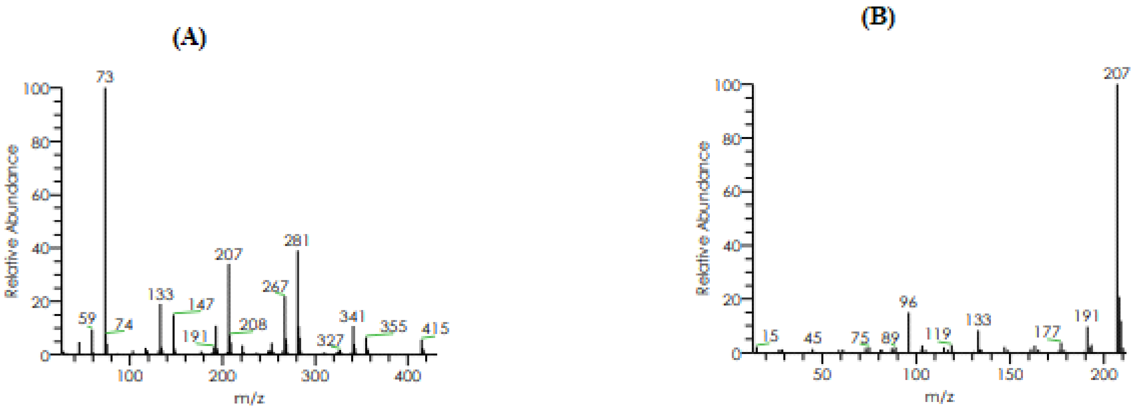

(A) Octasiloxane (B) Dimethylsiloxane cyclic trimer

Figure S9. Individual mass fragmentation of Each Compound.

Table S3. List of major components and their parameters identified in Fraction 3 of *Cydonia oblonga.*

Retention time (RT)	Molecular mass (g/mol)	Molecular formula	Area %	Area	Peak hight	Compound name
25.57	578	$C_{16}H_{50}O_7Si_8$	1.56	1288630. 27	86531.92	Octasiloxane
25.57	222	$C_6H_{18}O_3Si_3$	1.56	1288630. 27	86531.92	Dimethylsiloxane cyclic trimer

GC-MS analysis of Fraction 4

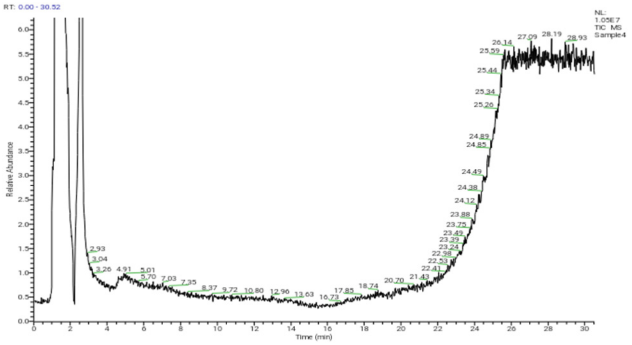

Figure S10. GC-MS chromatogram of Fraction 4 of *Cydonia oblonga.*

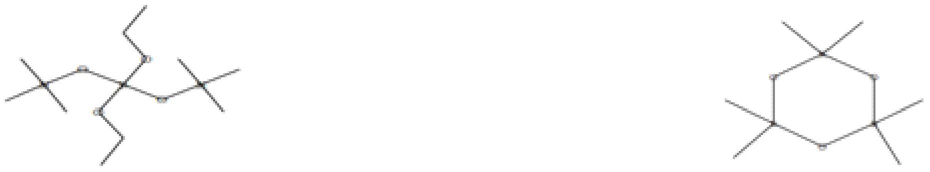

Silicic acid Cyclotrisiloxane

Figure S11. Major phytochemical compounds identified in fraction 4of *Cydonia oblonga.*

(A) **(B)**

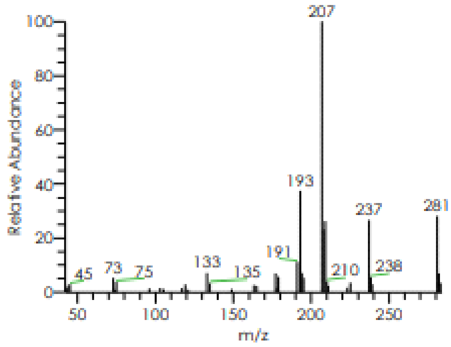

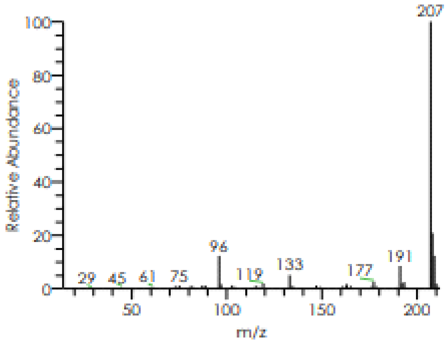

(A) Silicic acid **(B)** Cyclotrisiloxane

Figure S12. Individual mass fragmentation of Each Compound.

Table S4. List of major components and their parameters identified in Fraction 4 of *Cydonia oblonga*.

Retention time (RT)	Molecular mass (g/mol)	Molecular formula	Area %	Area	Peak hight	Compound name
25.67	296	$C_{10}H_{28}O_4Si_3$	0.94	1036227. 93	64734.42	Silicic acid
25.67	222	$C_6H_{18}O_3Si_3$	0.94	1036227. 93	64734.42	Cyclotrisiloxane

GC-MS analysis of Fraction 5

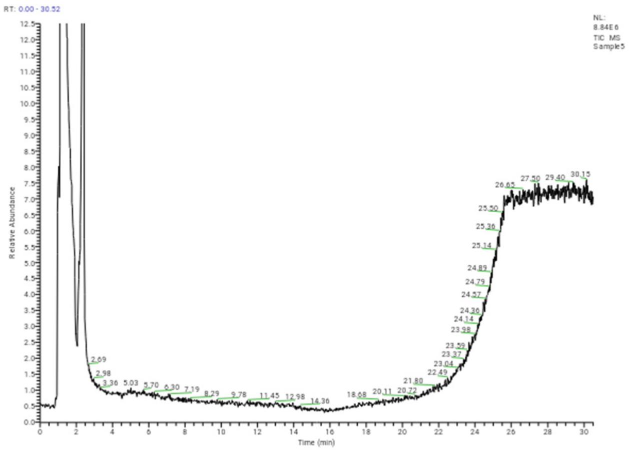

Figure S13. GC-MS chromatogram of Fraction 5 of *Cydonia oblonga.*

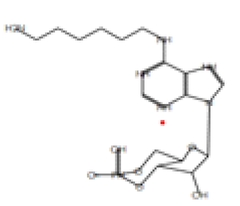

6-AH-cAMP

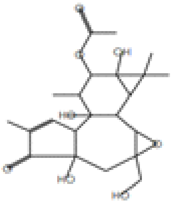

4H-Cyclopropa[5',6']benz[1',2':7,8]azule

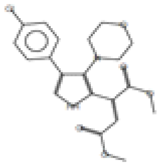

(4-(4-Chlorphenyl)-3-morpholino-pyrrol-2-yl)-

Figure S14. Major phytochemical compounds identified in fraction 5 of *Cydonia oblonga.*

Table S5. List of major components and their parameters identified in Fraction 6 of *Cydonia oblonga.*

Retention time (RT)	Molecular mass (g/mol)	Molecular formula	Area %	Area	Peak high	Compound name
25.61	247	$C_{16}H_{24}N_6O_6P$	0.61	573972.74	61425.84	6-AH-cAMP
25.61	422	$C_{22}H_{30}O_8$	0.61	573972.74	61425.84	4H-Cyclopropa[5',6']benz[1',2'.7,8]azule
25.61	404	$C_{20}H_{21}ClN_2O_5$	0.61	573972.74	61425.84	(4-(4-Chlorphenyl)-3-morpholino-pyrrol-2-yl)-butenedioic acid

GC-MS analysis of Fraction 6

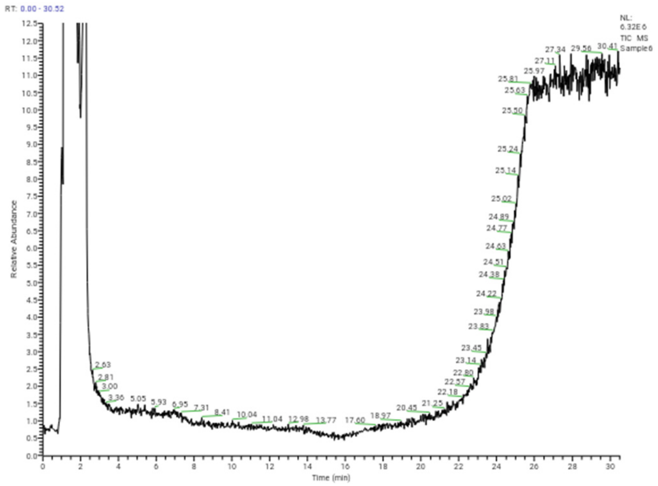

Figure S15. GC-MS chromatogram of Fraction 6 of *Cydonia oblonga.*

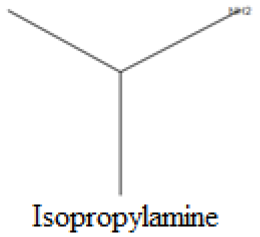

Figure S16. Major phytochemical compounds identified in fraction 6 of *Cydonia oblonga.*

(A)

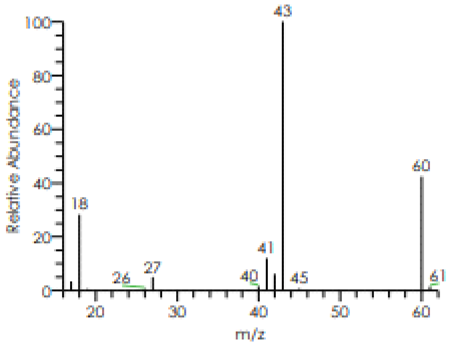

Figure S17. Individual mass fragmentation of the Compound. (A) Isopropylamine.

Table S6. List of major component and their parameters identified in Fraction 6 of *Cydonia oblonga.*

Retention time (RT)	Molecular mass (g/mol)	Molecular formula	Area %	Area	Peak hight	Compound name
2.30	59	C_3H_9N	27.59	28847904 .44	6067613.94	Isopropylamine

GC-MS analysis of Fraction 7

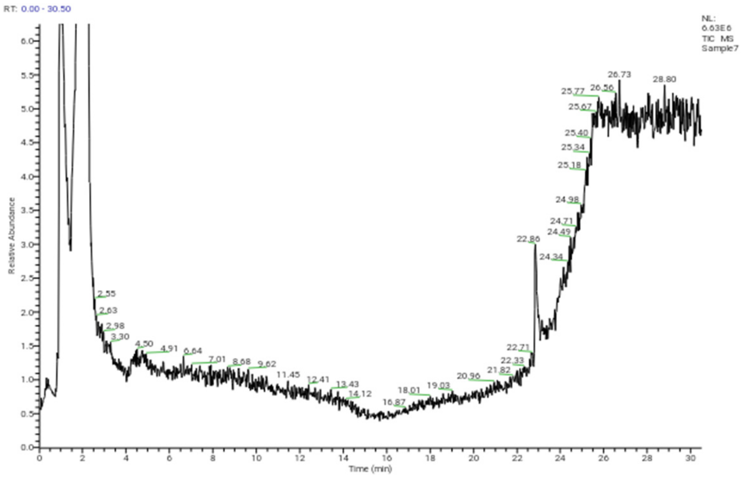

Figure S18. GC-MS chromatogram of Fraction 7 of *Cydonia oblonga*.

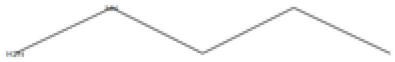

1-Propylhydrazine

Figure S19. Major phytochemical compounds identified in fraction 7 of *Cydonia oblonga.*

(A)

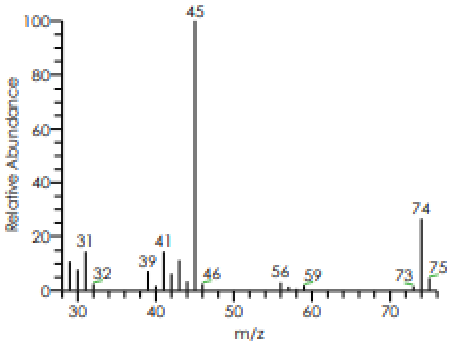

Figure S20. Individual mass fragmentation of the Compound. (A) 1-Propylhydrazine.

Table S7. List of major component and their parameters identified in Fraction 7 of *Cydonia oblonga*.

Retention time (min)	Molecular mass (g/mol)	Molecular formula	Area %	Area	Peak hight	Compound name
1.04	74	$C_3H_{10}N_2$	9.89	4594776. 26	303885.27	1-Propylhydrazine
